# Granuloma Annulare Located on Striae Distensae

**DOI:** 10.5826/dpc.1102a18

**Published:** 2021-04-12

**Authors:** Tal Goldberger, S. Sheffer Levi, Gil Armoni, Stephanie Ben-Shushan, Alexander Maly, Yuval Ramot

**Affiliations:** 1 Department of Dermatology, Hadassah Medical Center and the Faculty of Medicine, Hebrew University of Jerusalem, Jerusalem, Israel; 2 Department of Pathology, Hadassah Medical Center, Hebrew University of Jerusalem, The Faculty of Medicine, Jerusalem, Israel

**Keywords:** granuloma annulare, dermatopathology, striae distensae, scar

## Introduction

Granuloma annulare (GA) is a noninfectious granulomatous dermatitis that is more common in children and young adults. It manifests clinically as localized or generalized rash in micropapular, nodular, perforating, patch, or subcutaneous forms. Insect bites, trauma, tuberculin skin testing, ultraviolet radiation exposure, and bacterial and viral infections have been proposed as inciting factors; however, the exact pathophysiology of GA remains elusive [1].

## Case Presentation

**Figure 1:**
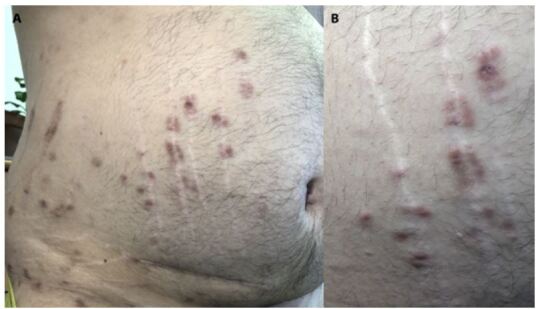
(A) Multiple erythematous, violaceous papules and nodules located on the abdomen of a man, distributed mostly along striae distensae. (B) A closer perspective of the lesions on the abdomen.

**Figure 2:**
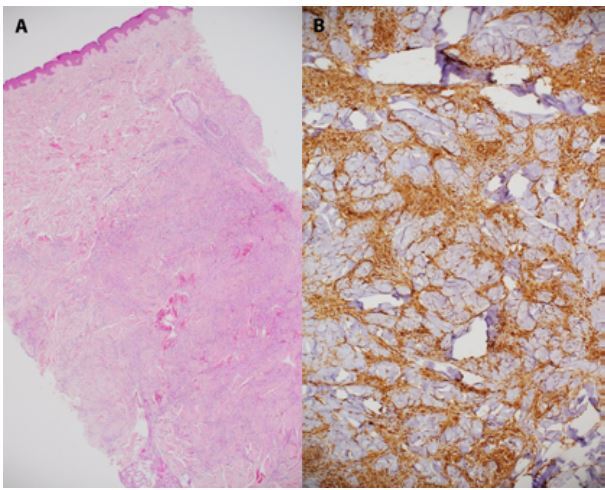
Histopathologic image of a skin biopsy taken from a lesion on the abdomen, demonstrating (A) diffusely infiltrated dermis with histiocytes and lymphocytes (H&E, original magnification ×40). (B) CD68+ histiocytes diffusely infiltrating the dermis (original magnification ×100).

A 36-year-old male with a history of diabetes mellitus, hypertension, hyperlipidemia, fatty liver, gout, nephrolithiasis, benign thyroid nodules, and glaucoma presented to our outpatient dermatology clinic with nontender papulonodular lesions of 1 year’s duration. Skin examination revealed purple-brown papules and nodules distributed symmetrically on the abdomen with accentuation and accumulation of the lesions mostly over areas of striae distensae (SD), and to a lesser degree on the thighs (Figure 1, A and B). Punch biopsies taken from the abdominal lesions demonstrated a diffuse inflammatory infiltrate, composed mainly of CD68+ histiocytes and lymphocytes (Figure 2, A and B). These findings were consistent with incomplete GA.

## Conclusions

Granuloma annulare is known to be diverse in morphology, distribution, and histology. Yet, the unique distribution pattern of the lesions in our case, localized mainly to the SD, was intriguing. Lesions of GA were reported to occur at sites of previous skin conditions, such as herpes zoster, and at sites of trauma and inflammation, such as following vaccinations, tattoos and surgical incisions [2].

Striae distensae have been previously associated with several skin lesions, including leukemia cutis, keloid, linear focal elastosis, urticarial vasculitis, lupus erythematosus, chronic graft-versus-host disease, and a granulomatous reaction following microneedling. The formation of striae alba (SA), the mature scar-like form of SD, is accompanied by several immune events that include mast cell degranulation, macrophage activation, perivascular lymphocytic cuffing, and dermal edema. These immune events eventually allow the maturation (healing) of the primary erythematous striae rubra into the pale, atrophic, scar-like SA. The various immune processes involved in the formation of SD may, in susceptible patients, recruit T-helper and other immune cells to promote the formation of GA in the context of aberrant wound healing [2].

Our case suggests a possible connection between GA and SD. Previous reports have associated the formation of GA with scarred or irritated skin. Therefore, the development of GA in the distribution of SD, which is a phenomenon of dermal scarring, is biologically plausible.
